# News coverage of drug development: implications for the conveyance of health information

**DOI:** 10.1186/s12889-021-11849-8

**Published:** 2021-10-07

**Authors:** Jiangtao Wang, Wing-Fu Lai

**Affiliations:** 1 Section of Science, Southern Weekly, Guangzhou, China; 2 School of Life and Health Sciences, The Chinese University of Hong Kong (Shenzhen), Shenzhen, 518172 China; 3 School of Education, University of Bristol, Bristol, UK

**Keywords:** Health information conveyance, Chinese news media, Illusion of knowing, Drug development

## Abstract

**Background:**

Technical information regarding health-related advances is sometimes esoteric for the general public. News media, therefore, plays a key role in public health promotion via health information conveyance. In this study, we use China as a sample country and analyze the claims and frames in news coverage of health-related advances, with special focus on news coverage of the development and performance of newly developed or tested drugs.

**Methods:**

A keyword search was performed to retrieve news articles from four representative news agencies in China. In total, 3029 news reports were retrieved, of which 128 were selected for further analysis.

**Results:**

Four aspects of news coverage of drug development were identified: (1) the characteristics of new drugs covered, (2) the sources of information, (3) the accuracy of health information in newspapers, and (4) textual features of news coverage.

**Conclusions:**

Our findings reveal that guidelines should be established to facilitate more systematic news reporting on health-related advances. Additionally, literacy among the general public and professionalism in health information conveyance should be promoted to negate the “illusion of knowing” about health-related advances.

## Background

Drug development promotes human health, and the number of newly launched drugs is negatively related to mortality [1]. According to data collected from 36 countries in 2015, the cancer disability-adjusted life-years (DALYs) for 19 types of cancers have reduced by almost 23% because of new drugs launched between 1982 and 2010 [2]. Estimated mortality also substantially reduced between 2015 and 2020 because of new drug development between 2011 and 2015 [2]. Drug development is, therefore, pivotal to public health promotion. However, immense investment in terms of technologies, resources, and time is required to develop just one drug [3]. In practice, the cost of developing a drug may exceed $800 million [4–6]; the high failure rate of drug development accounts for such high cost. Approximately 11.8% of new drugs are approved for clinical use [7]. Furthermore, 10 years or more are usually required to complete the drug development and evaluation process [8, 9].

Drug development is time-consuming and labor-intensive. Additionally, information about the research and development of new drugs is sometimes esoteric for the general public [10]. Therefore, news media plays a key role in public health promotion by enhancing public comprehension of the development, effects, and performance of new drugs [11]. A previous study has shown that people who closely follow health news are more likely to acquire correct health-related knowledge [12]. Additionally, news coverage enables the general public to make informed decisions regarding health care [13]. In reality, drug development constitutes a professional “biomedicalization” sphere, yet news media can help break such sphere boundaries and move health-related information from the restricted sphere to public sphere [10]. However, it is noteworthy that, in reality, news media may not positively contribute to enhancing public comprehension of drug development. Instead, under different frames of news coverage, public opinion about health-related technologies and advances could be shaped differently [11]. Accurate, balanced, and complete coverage of new health products is indispensable for helping the general public make informed and correct health decisions [13].

Despite this, previous research on news coverage of drug development has found that news reports often lack important and basic information about the drugs developed [14]. The content analysis of coverage of the human papillomavirus (HPV) vaccines in newspapers in the US has also shown that even detailed information on newly developed drugs is frequently incomplete [15], with various types of essential information (including the experimental status, drug efficacy, and mechanism of drug action) missing. A recent study of 500 stories in the US media revealed that the incompleteness of information reported in news articles was mainly associated with the risks and cost of the product and treatment [13]—more than 50% of news coverage omitted the potential treatment risks, and 70% did not reveal the treatment cost [13]. This may have a negative effect on people’s medical decisions. Therefore, quality evaluation of news coverage of health-related technologies and medicines is important.

Currently, the majority of studies on news coverage of drug development and its public health implications are conducted in the US [10, 13] and other high-income countries [16]; few such studies are, or have been, conducted in developing countries. However, the transferability of findings from the US and other high-income countries to developing countries is a worthwhile concern. This is partially supported by the fact that claims and frames in news coverage of drug development can be affected by cultural factors. In the case of Kalydeco (a drug for cystic fibrosis), a Canadian newspaper emphasized the availability of the drug under the public funding program for patients, whereas news coverage in the US framed the drug as an economic story [17]. Similarly, in the case of trastuzumab (a drug for breast cancer), news coverage in both Canada and the UK presented it in a positive light, but the tone of the Canadian newspapers was more neutral [18]. Thus, diverse narratives can be constructed around the same drug through different styles of news coverage.

Examining news coverage in developing countries is important because: (1) they generally have a poor system of regulating the quality and accuracy of science news, and (2) the comparatively low educational level of people in these countries makes them susceptible to misleading or scientifically inaccurate information. Therefore, the objective of this study is to analyze the news coverage of drug development in the main health newspapers in China. Strict regulations regarding the accuracy of new drug information (as well as the professional qualification of health reporters) in news coverage are lacking in China. Consequently, it is a good sample country that offers a suitable environment to study implications of improving the quality of news coverage of drug development to enhance public comprehension of advances in drug development for public health promotion. In order to achieve this goal, the following research questions are answered:

1. What are the characteristics of the new drugs reported by the Chinese news media?
2. What are the textual features of news reports on new drugs in China?
3. How accurate is the drug information reported by the Chinese news media?

## Methods

As heterogeneous information (ranging from topic-choosing to linguistic expression) exists in news coverage, a deep content analysis can help reveal the complexity of new drug information. In order to analyze the claims and frames in Chinese news coverage of drug development, four influential newspapers: *Science and Technology Daily*, *Health News*, *Zhongguo Yiyao Bao*, and *Medicine Economic Reporter* were included in this analysis. All of them are managed by national government agencies of China: *Science and Technology Daily* is managed by the Ministry of Science and Technology of the People’s Republic of China; *Health News* is managed by the National Health Commission of the People’s Republic of China; and *Zhongguo Yiyao Bao* and *Medicine Economic Reporter* are managed by the National Medical Products Administration. They are issued countrywide because of their special and nearly irreplaceable roles in reporting drug development. They report profusely on drug development and set the tone for relevant drug information, because of which they are acknowledged as mainstream news media by the China News of Drug Information Association and as major resources of health information in China [19]. The China Core Newspapers Full-text Database (CCND) of the China National Knowledge Infrastructure (CNKI) was used to search for relevant news reports [20, 21]. A database search for the period 2015–2020 with the Chinese equivalent of the keyword “new drug,” revealed that the number of news reports published in *Science and Technology Daily*, *Health News*, *Zhongguo Yiyao Bao*, and *Medicine Economic Reporter* was much higher than that published in other newspapers. As a result of the social interruption caused by COVID-19, news coverage of new drugs in 2020 was atypical and non-representative. Therefore, only news reports published between 2015 and 2019 were retrieved.

**Fig. 1:**
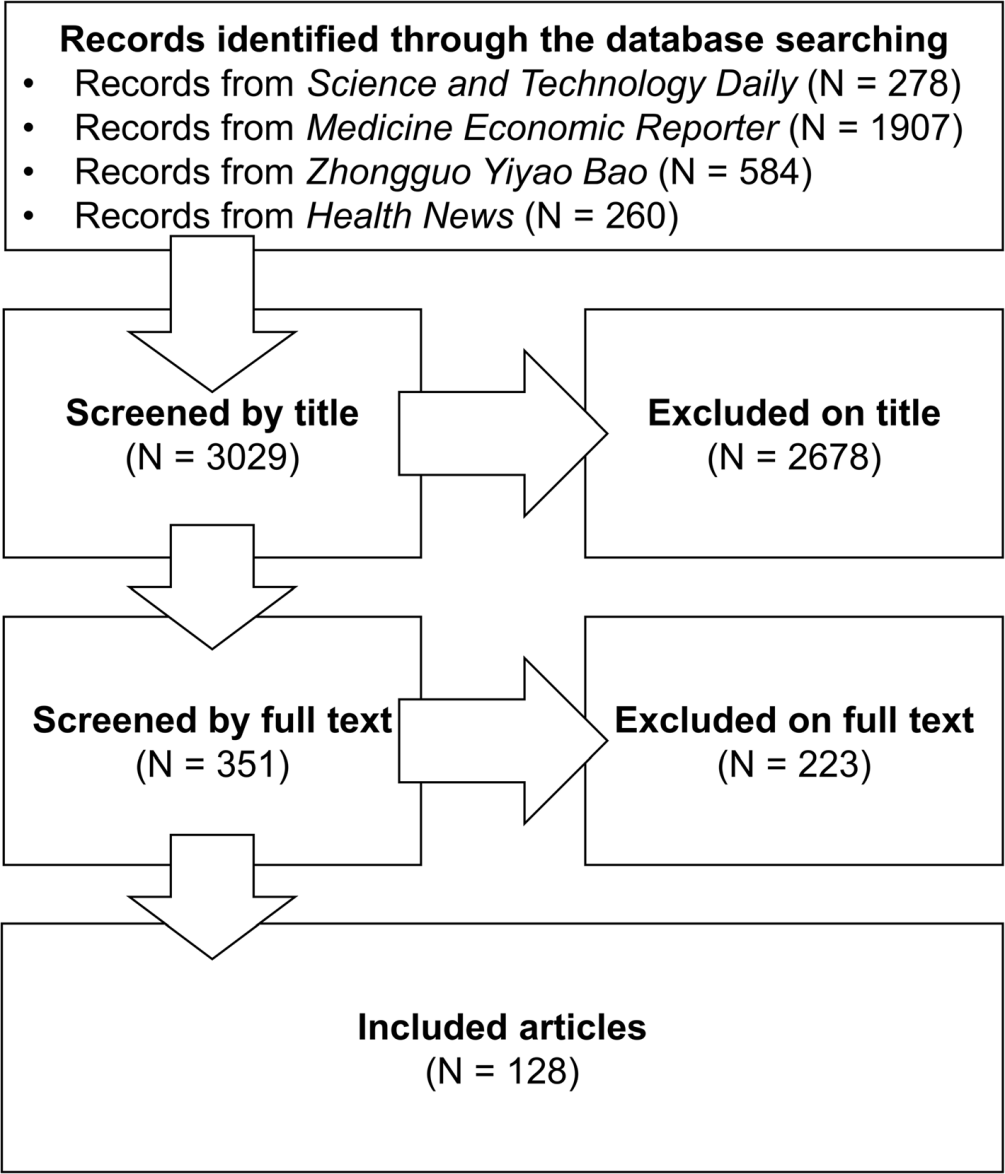
Flow diagram depicting the process of article screening and selection

**Fig. 2:**
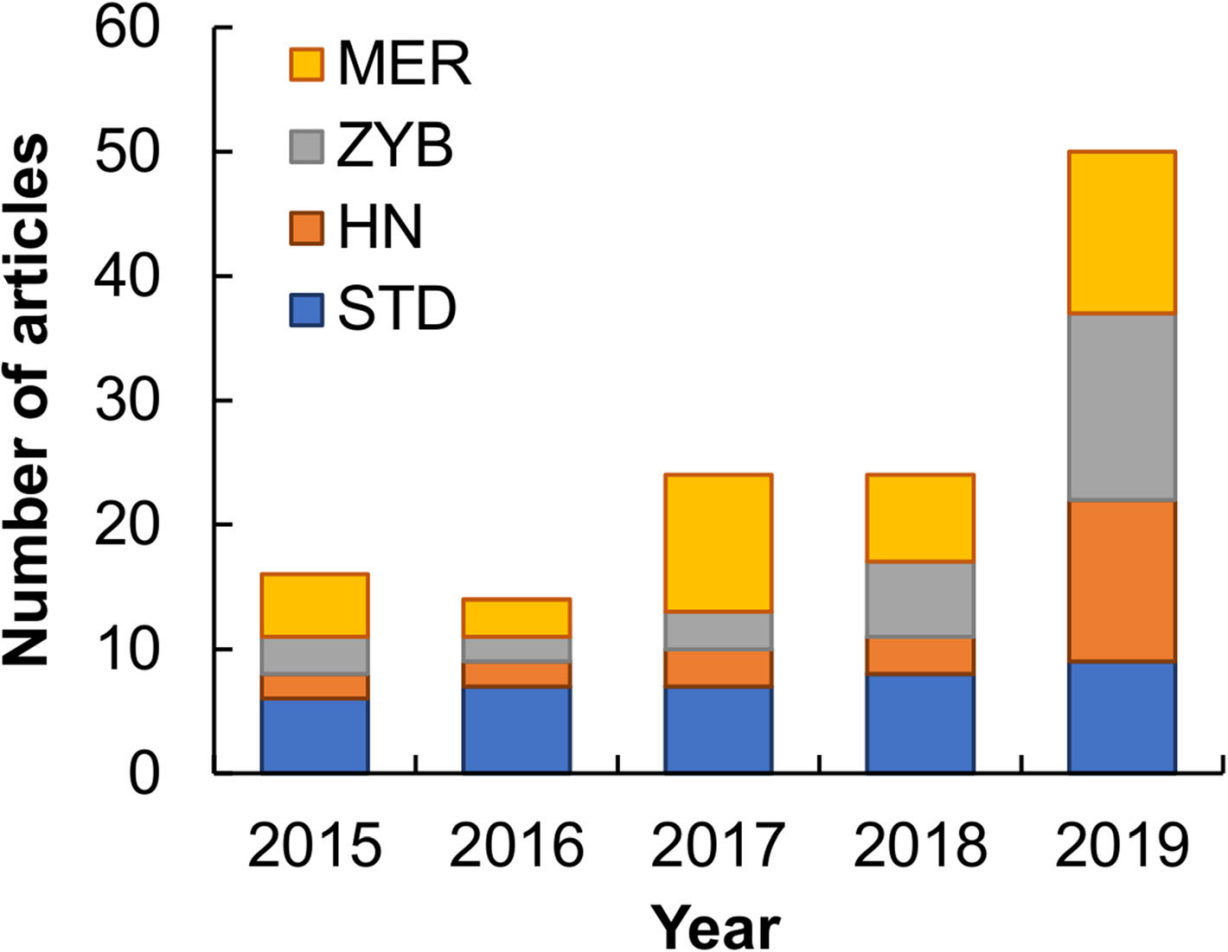
Number of articles retrieved from 2015 to 2019. Abbreviations: STD, *Science and Technology Daily*; MER, *Medicine Economic Reporter*; ZYB, *Zhongguo Yiyao Bao*; HN, *Health News*

A total of 3029 news reports were retrieved, but only those that introduced the research, development, or approval of at least one new drug were further analyzed. The news reports were independently screened by both authors, and inter-rater reliability was assessed using Cohen’s kappa, which was calculated to be 0.82 and considered good agreement beyond chance. All disagreements encountered during the news report selection process were discussed until consensus was reached. A total of 128 news reports met the inclusion criteria and were used for further analysis (Fig. 1**,** Table 1). Among them, 37 reports were from *Science and Technology Daily*; 39 from *Medicine Economic Reporter*; 29 from *Zhongguo Yiyao Bao*, and 23 from *Health News* (Fig. 2). Each article was analyzed from three aspects. The first aspect was related to the characteristics of new drugs reported in mass media, and the function, development phase, and place of origin of the reported drug were analyzed. The second aspect was related to the features of news coverage, with special focus on the reporting style and the information source of the news report. The last aspect was related to the accuracy of the content in news reports, and the availability of information about new drug mechanisms and presence or absence of misleading information about the reported drug were examined.

**Table 1:** Number of articles excluded on the basis of different reasons

Reason	Number of articles	
	Title screen	Full-text screen
Articles reporting mainly the general progress and/or direction of drug development and biomedical research	453	42
Articles reporting mainly the development and achievement of a drug company or institution	281	13
Articles reporting mainly political issues	985	64
Articles reporting mainly the investment and market dynamics in drug development	847	88
Articles reporting mainly treatment development in which drugs are not the focus	59	13
Articles reporting mainly the story of a person	43	2
Articles reporting mainly the general understanding of a disease	10	1

## Results

### Characteristics of newly developed drugs covered in Chinese newspapers

Among the 128 news reports analyzed, 23.4% (*N* = 30) were related to cancer treatment. The second and third most frequently covered diseases included infectious (*N* = 29, 22.7%) and neurodegenerative (*N* = 18, 14.1%) diseases, respectively. Alzheimer’s disease (AD) was the most frequently mentioned neurodegenerative disease. In total, 13 articles covered new drugs developed for the treatment of AD. Other diseases that have been covered fairly frequently include renal anemia (*N* = 9, 7.0%), type 2 diabetes (*N* = 8, 6.3%), and AIDS (*N* = 7, 5.5%). Diseases addressed by the drugs in the analyzed news reports are listed in Table 2. The reporting on the developmental phases of new drugs is heterogeneous. Among the news reports analyzed, 57.8% (*N* = 74) reported new drugs with approved or nearly approved status. Reports on new findings on drugs in clinical trials ranked behind at 28.1% (*N* = 36), followed by reports on new drugs in pre-clinical trials (*N* = 18, 14.1%). Among the new drugs reported, most address diseases that are highly prevalent in China. This is consistent with a previous finding that rare diseases are more likely to be ignored by news media compared to prevalent diseases [22].

**Table 2:** Diseases tackled by drugs reported in the analysed news articles

Disease	Number of articles	Percentage
Cancer	30	23.4%
Infectious disease	29	22.7%
Neurodegenerative disease	18	14.1%
Immunological disease	13	10.2%
Kidney disorder (except cancer)	11	8.6%
Type 2 diabetes	8	6.3%
Cardiovascular disease	6	4.7%
Neuropsychiatric disorder	3	2.3%
Bone rarefaction	2	1.6%
Miscellaneous	10	7.8%

An analysis of the location of the main researchers or developers of new drugs mentioned in news reports revealed China as the most frequent location. A total of 57% of the analyzed news articles reported new drugs mainly or predominately developed in China. Several reports mentioned the quick review and approval process for new drugs by the Chinese authorities; this can reveal the issue of drug administration and policy changes in China. In fact, prolonged review time once caused a delay in drug approval in China, which led to the drug registration system reform in 2015 [23]. More than half of the analyzed reports were about domestic drugs, which conveys a strong nationalist standpoint on new drug coverage in Chinese newspapers.

Apart from China, other locations where the reported drugs are developed include the US (32.8%) and the UK (10.9%). Companies and/or universities in various countries (including Switzerland and Japan) are also involved in the development of drugs reported by mass media in China. Here, it is noteworthy that the high percentage (57%) of analyzed articles reporting new drugs mainly or predominantly developed in China may also be attributed to the fact that the analyzed newspapers are published in Chinese and circulated mainly in China. Therefore, most drugs reported by the mass media in China tend to be those more relevant to Chinese people or the local market, even though more new drugs are developed in the US and European countries [24]. Nevertheless, approximately one-third (32.8%) and one-fourth (24.2%) of the analyzed reports covered new drugs developed by US and European companies, respectively.

### Sources of new drug information

Information sources constitute the news coverage skeleton. Pharmaceutical companies were the foremost information source for news coverage of news drugs in China. A total of 36.7% of news reports analyzed included information provided by members of pharmaceutical companies, and 21.9% directly quoted information provided by pharmaceutical companies. Drug researchers constituted the second major source of information; approximately 31.3% of the analyzed news reports relied on them. Articles published in academic journals were also cited as an information source by 14.1% of the analyzed reports. Other sources of information accessed by the mass media included governmental agencies (12.5%), physicians (8.6%), news briefings (8.6%), academic conferences (7.8%), and officials from drug regulatory bodies (7.8%). The full list of information sources used by the analyzed reports is listed in Table 3.

**Table 3:** Information sources used by the analyzed news reports

Information source	Number of reports	Percentage
**Human source**		
Pharmaceutical company member	47	36.7%
Researcher	40	31.3%
Physician	11	8.6%
Governmental official	10	7.8%
**Non-human source**		
Pharmaceutical company	28	21.9%
Journal article	18	14.1%
Regulatory body	16	12.5%
Press conference	11	8.6%
Academic conference	10	7.8%

Public perception of new science could be a complex process based on information from many sources besides news media [25]. Some of these sources include governmental agencies [26] and academic journals [27]. Academic articles and press releases from governmental agencies are two sources that provide comparably precise information about new drugs; however, compared to pharmaceutical companies, these two sources were used in less than 30% of the analyzed news reports. Importantly, even when news reports used information from these two sources, research limitations and safety warnings were rarely mentioned. Previously, a study that analyzed news coverage of health-related information in the US from the 1960s to 2000s found that diverse sources (including researchers and patients) of health information were used by the mass media [10]. This is slightly different from our findings, in which the diversity of sources of information in the Chinese media is relatively low. In addition, none of the analyzed news reports treated patients as a source, and more than half (51.6%) of the reports included unclear sources, that is, much of the provided information was cited as news content, but the sources were not revealed. These news reports usually contained phrases such as, “*It is learned that …*” [28], “*according to the information …”* [29], “*according to the introduction …”* [30], and “*experts say …”* [31]. From the textual perspective, this information was clearly not from the reporters, but it was difficult to infer the source.

### Accuracy of scientific knowledge in newspapers

Although some news reports delineated information about the mechanisms of new drugs, approximately 60% of the reports did not. Among the reports describing the drug mechanism, most of the reported drugs were in the early developmental phase. Specifically, for new drugs in pre-clinical trials, 72.2% of the analyzed articles provided a further introduction to the drug mechanism. For drugs in clinical trials, the percentage of reports mentioning the drug mechanism dropped to 41.7%. This percentage was further reduced to 35.1% for drugs approved or nearly approved for clinical use. This may be due to the fact that when a drug is endorsed by trustworthy agencies [32] or approved by governmental bodies, there is less uncertainty about its effect, which may shift the focus of the reports from why the drug works to how well the drug works. It is worth mentioning, however, that descriptions of the drug mechanism may usually be accompanied with information about the uncertainty involved with the new drug. A lack of such information may influence the public risk perception of the reported drug.

Additionally, 23.4% of news reports contained information unrelated to the reported drugs *per se*. When the drugs developed in China were concerned, the percentage of reports containing irrelevant information was around 30.6%. This percentage dropped to 12.7% when the drug reported was developed overseas. An example of such irrelevant information includes information about the company that manufactures the reported drug. This can be partially demonstrated by the following excerpts.

R2: “*The company XXX chose products with high market potential to optimize the drug delivery route*.”

R18: “*Innovation is the inexhaustible impetus for enterprises’ development and is the foundation for company XX to survive on. In 2008, person XXX set up company XX in cooperation with another company after returning from overseas and engraved innovation into the soul of the enterprise.*”

R42: “*The company XX had brought Chinese patients several world-leading drugs within only two years...the approval of the drug XXX in China shows the promise of the company XX to Chinese patients.*”

Some news reports emphasized the sophistication of new drug research, development, and administration in China. This is exemplified by the excerpt provided below.

R19: “*The good news about the approval of the drug XXX by the FDA made us extremely proud! This historic breakthrough not only represents the international endorsement of our national new drug development … it proved our innovative pharmaceutical companies could not only benefit the patients in our country but also have sufficient strength to serve more patients worldwide.”*

Additionally, new drug information is sometimes linked to the success of pharmaceutical entrepreneurs. This is shown in a news report as follows:

R34: “*The success of the drug XXX brought the company and the chairman a lot of honors. Its success was recognized as a domestic entrepreneurship model.”*

It is noteworthy that adding information about the companies may deprive the report of its independence from commercial considerations. Whenever unrelated information appeared in reports, as mentioned above, it was always presented positively. This not only puts the reported drug in an unfairly positive light but also may mislead the public regarding the drug. Therefore, conflicts of interest should be disclosed to enhance the quality of news coverage. Unfortunately, such disclosure statements were rarely found in the analyzed reports.

### Textual features of news coverage of new drugs

Only 9.4% (*N* = 12) of the analyzed news reports mentioned the risks of the reported drugs, with the majority of the reported risk information being related to adverse drug reactions (e.g., nausea, tiredness, and headache) found in clinical trials. The remaining articles (*N* = 116, 90.6%) failed to include any information on the risks or harm of the reported drugs. A similar practice of omitting risk disclosure in news coverage of new drugs has been observed in various previous studies [33–35]. However, the percentage (90.6%) in case of the Chinese media is substantially higher than that found in the Canadian newspapers (68%) [33] and the US media (53%) [35]. After a drug reached the approval stage, 90.5% of the analyzed news articles failed to mention any potential risk, describing only the positive aspects. In addition, 57% of the analyzed news reports used adjectives such as “best,” “first,” and “only” to describe the performance and importance of the reported drug. In case of articles reporting drugs in the approval stage, the percentage of reports using absolute adjectives increased to 73%. Thus, emphasizing the benefits of the reported drugs and hiding their potential risks were the two textual features of news reports on new drugs. This can be partially demonstrated through the example of a news report on a new hepatitis B drug. The reporter described the drug as an “*unprecedented hepatitis B drug with the best anti-virus efficiency*” [36]. Other examples obtained from news reports described drugs for chronic obstructive pulmonary disease and systemic lupus erythematosus.

R93: “*No product in the market can compare with it...the first formulation for chronic obstructive pulmonary disease in the world, and the only drug that can reduce all-cause mortality by decreasing the admission rate of patients with chronic obstructive pulmonary disease.*”

R102: “*This is the first time … for an original drug developed in China to be invited to present a report at the annual conference of the American College of Rheumatology... the drug XXX shows promise to become the world’s first multi-target drug to tackle systemic lupus erythematosus.*”

The use of absolute adjectives to emphasize the positive aspects of a drug while omitting its negative effects raises concerns about the neutrality of the Chinese mass media. The provision of only positive information about a reported drug suggests that the media is uncritical and accepts information about a drug without questioning [37]. The textual features of news coverage of new drugs developed in other countries are different. When the reported drug was developed in China, 95.2% of the reports did not disclose risks and 66.1% of them used absolute adjectives; however, when the reported drug was developed overseas, these percentages dropped to 83.6 and 41.8%, respectively. In fact, the efficacy of a drug may decline over time, especially after a period of clinical use. Mass media-fueled optimism about the power of a new drug may hinder accurate public perception of the actual potential of the reported drug, thereby leading to the illusion of knowing [38].

## Discussion

Exploring how newly developed or tested drugs are reported by newspapers—as well as the textual features and factual accuracy of those reports—can help comprehend their possible impact on the public’s perception of the reported drugs. Earlier studies on health news coverage in developed countries have not only observed that reports on new drugs are often written based on a very limited variety of information sources [14], but have also found that many of the reports fail to disclose essential information (including the efficacy and mechanism of the reported drug [15]) and to offer a balanced account of the risk and benefit of using the drug [13]. This is consistent with the observation we made in this study. As shown by the results, more than half of the analyzed news reports employed adjectives such as “best,” “first,” and “only” to describe the performance and importance of the reported drugs. More than one-fifth (21.9%) of the reports directly quoted the information provided by pharmaceutical companies. News reports using information collected from independent experts and patients are highly scant. In addition, over 90% of the reports failed to present a balanced account and focused solely on the positive aspects of the reported drug without mentioning any risks or harm warnings; the rate was higher than that in Canadian newspapers (68%) [33] and the US media (53%) [35]. This may be attributed to the reporting principles established by China for media management, which requires the news media in China to report more positive aspects of social development so as to keep the people and the country “united,” “stable,” and “motivated” [39].

In order to ensure a more balanced and objective description of the reported drug-related information in mass media, the establishment of guidelines on areas to be reported may help improve the overall quality of reporting in health-related domains. Important aspects that may affect the standpoint and/or reveal personal interests of the news reporter (including the source of information), as well as basic information about the drug or related treatment (including the scope of usage, known and potential adverse effects, and the manufacturer’s name) should be specified. The availability of alternative treatment options, as well as the cost of the reported drug, should also be covered in a news report [40]. Additionally, miscomprehension of drug efficacy may impede accurate health decision-making [41]. Our results show that most of the diseases reported in the analyzed news reports included common chronic diseases prevalent among a large patient population. Extensive use of absolute adjectives such as “best,” “first,” and “only” without providing risks disclosure can have a negative public health impact. Therefore, the use of adjectives should be regulated through carefully created guidelines.

After the establishment of guidelines, their implementation should be closely monitored by professional bodies related to science journalism so as to assure that their members adhere to the guidelines. In order to achieve this goal, regular assessment of news quality, as well as systems for reward and punishment, may be put into effect. Additionally, when new drugs and other medical treatments are reported, the voices of different stakeholders (including physicians, patients, and scientists) should be reflected as much as possible to ensure that the content of the news report is objective, comprehensive, accurate, and unbiased. Unsupported claims in some critical medical areas should be prohibited by law, just as claims on health and therapeutic benefits for foods and nutritional supplements are legally prohibited.

In order to promote accurate public perception of health-related information, school students should be trained to acquire critical analysis skills through the incorporation of assignments and assessment tools requiring critical thinking into the curriculum. From a broader perspective, public health promotion should be enhanced so that the mass media is no longer the only information source and voice. If the general public can access multiple sources of health information, they will judge the accuracy of the reported health-related advances based on information retrieved from diverse sources. This can help avoid the “illusion of knowing” [42]. In fact, improper reporting practices can easily promote the illusion of knowing [43], causing people to possess less factual knowledge than they may perceive. This is particularly true for those who make prospective judgments or possess a low level of academic achievement [44], and they are more susceptible to the illusion of knowing. Therefore, raising literacy among the general public will be an important step to promote accurate public comprehension of health-related advances and, hence, public health as a whole.

In addition to literacy among the general public, health reporters play an important role in enhancing the public’s understanding of health-related advances. Therefore, they could adopt a more public-oriented approach while drafting a news report. In this study, we found that the patient was not cited as an information source in any of the analyzed news reports. The responsibility of mass media is to disseminate useful information to the public; therefore, journalists are expected to report the news in ways that reflect and fulfill the needs of society [40]. As far as news reports on clinically tested health-related advances are concerned, they link inextricably to the needs of patients. Voices and experiences of patients should be presented when health-related messages are conveyed to the general public [45]. Furthermore, because public attention is limited and difficult to sustain, an over-detailed description of a new drug or treatment may cause readers to lose interest [46], but an oversimplified or even biased account may cause the illusion of knowing. Journalists are, therefore, required to seek a point of balance. Owing to the technical aspect of news reporting, it would be best if news reports on health-related advances are written by journalists with educational training in related health disciplines [47]. Qualified journalists should possess professional knowledge, access appropriate primary sources of health information, and exhibit the ability to analyze public health issues in a neutral manner [48]. Additionally, journalistic professionalism should be maintained via the continuing professional development (CPD) system, which has already been recognized as an integral part of career development for health professionals [49], teachers [50], and journalists [51]. The ever-changing public-health arena renders the CPD system indispensable for journalists to be able to fully perform their duties in promoting public comprehension of health-related advances.

## Conclusions

News media plays an important role in promoting public comprehension of health-related advances, which, in this study, were constricted to the progress in drug development. Over the years, considerable effort has been devoted to examining the mass media in developed countries [10, 13–15, 52, 53], but few such studies have been performed in the context of developing countries. This study addresses this gap using China as a sample and analyzing the claims and frames in news coverage to ascertain how advances in drug development are depicted in news reports in a developing country. It is noteworthy that all our findings are based entirely on our analysis of news reports from representative media agencies in China, where state-owned news media dominate the news dissemination space. In view of the cultural differences among countries, the conclusions and recommendations made in this study may not necessarily be directly translatable to other countries. Moreover, over the last several decades, the influence of news coverage on public health has been shaped by changes in the pattern of information access [54–57]. Such changes are led by the increasing diversity of media technologies [58]. Apart from traditional news media, a growing number of people use social media to obtain information [59, 60]. National-level surveys reveal that 25.5% of people in China read newspapers [61] while 77.1% read news reports online [62]. The influence of social media on news reading habits increases the complexity of drug information communication [63]. However, the present study focused only on news media. The impact of social media on conveyance of drug information for public health promotion may be worth examining in future studies in order to attain a comprehensive picture of the influence of news coverage on public health. Nevertheless, news media in China serves as a “mouthpiece” of the nation and of the Communist Party of China [63], which constitute the dominant voices. Conversely, social media serves only as a side source of information. Therefore, examining the news coverage of drug development advances in Chinese mass media can suggest strategies to facilitate accurate public perception of health-related issues.

## Data Availability

The data used and/or analysed during the current study are available from the corresponding author on reasonable request.

## Abbreviations

DALYs: Disability-adjusted life-years
CCND: China Core Newspapers Full-text Database
CNKI: China National Knowledge Infrastructure
HPV: Human papillomavirus
AD: Alzheimer’s disease
CPD: Continuing professional development
